# A recombinant vesicular stomatitis-based Lassa fever vaccine elicits rapid and long-term protection from lethal Lassa virus infection in guinea pigs

**DOI:** 10.1038/s41541-019-0104-x

**Published:** 2019-02-08

**Authors:** Derek R. Stein, Bryce M. Warner, Geoff Soule, Kevin Tierney, Kathy L. Frost, Stephanie Booth, David Safronetz

**Affiliations:** 1 Zoonotic Diseases and Special Pathogens, National Microbiology Laboratory, Public Health Agency of Canada, Winnipeg, MB Canada; 2 Department of Medical Microbiology, University of Manitoba, Winnipeg, MB Canada

## Abstract

The World Health Organization has identified Lassa virus (LASV) as one of the top five pathogens to cause a severe outbreak in the near future. This study assesses the ability of a leading vaccine candidate, recombinant Vesicular stomatitis virus expressing LASV glycoprotein (VSVΔG/LASVGPC), and its ability to induce rapid and long-term immunity to lethal guinea pig-adapted LASV (GPA-LASV). Outbred guinea pigs were vaccinated with a single dose of VSVΔG/LASVGPC followed by a lethal challenge of GPA-LASV at 7, 14, 25, 189, and 355 days post-vaccination. Statistically significant rapid and long-term protection was achieved at all time points with 100% protection at days 7 and 14 post-vaccination. While 83 and 87% protection were achieved at 25 days and 6 months post-vaccination, respectively. When guinea pigs were challenged one year after vaccination 71% protection was achieved. Notable infectious virus was isolated from the serum and tissues of some but not all animals. Total LASVGPC-specific IgG titers were also measured on a monthly basis leading up to LASV challenge however, it is unclear if antibody alone correlates with short and long term survival. These studies confirm that a single dose of VSVΔG/LASVGPC can induce rapid and long-term protection from LASV infection in an aggressive outbred model of infection, and supports further development in non-human primates.

## Lassa virus: Recombinant VSV Lassa virus vaccine

Lassa virus (LASV) is an emerging pathogen that can be associated with high case fatality but for which no clinically-approved vaccine currently exists. David Safronetz and colleagues at the Public Health Agency of Canada and the University of Manitoba investigate the efficacy of a single dose of a recombinant vaccine of LASV glycoproteins vectorized into vesicular stomatitis virus (VSVΔG/LASVGPC). Using guinea pigs lethally challenged with LASV, the protective efficacy of VSVΔG/LASVGPC and LASV-specific IgG is assessed at a number of time points out to approximately one year after vaccination. VSVΔG/LASVGPC elicits stable LASV glycoprotein-specific antibody production and durable protection from lethal LASV challenge, with 71% of animals surviving even at one year following vaccination and complete protection being afforded at earlier (weeks) time points. This pre-clinical model demonstrates the stable protection that can be established by a single dose of VSVΔG/LASVGPC.

## Introduction

In December 2015, the WHO convened an expert panel to prioritize the top five emerging pathogens that are likely to cause severe outbreaks in the near future. Of the top five pathogens, Lassa virus (LASV) was identified as a priority for accelerated research and development of vaccines for which none are currently available. The newly created Coalition for Epidemic Preparedness Innovations (CEPI) has also announced significant funding for LASV vaccine research. LASV is a rodent-borne *Mammarenavirus* which utilizes the multi-mammate rat, *Mastomys natalensis*, as its natural reservoir.^1^ Humans are frequently exposed to LASV following direct contact with virus laden-excreta from infected rodents or through close contact with infected humans/specimens. In humans, LASV infection can present with a wide spectrum of manifestations ranging from relatively asymptomatic or non-descript indictors of disease to severe hemorrhagic fever with multi-organ failure, a condition referred to as Lassa fever (LF).^2^ Estimates vary, though it is generally thought that between 300,000 and 500,000 people are infected with LASV on an annual basis, primarily in Sierra Leona, Liberia, Guinea and Nigeria making LASV one of the most prominent etiological agents of hemorrhagic fever worldwide.^2–5^ Although the fatality rate is considered to be between 1 and 2% of all infections, it increases dramatically in nosocomial settings and outbreaks where mortality rates exceeding 50% have been documented.^2,5,6^

The Vesicular stomatitis virus (VSV) recombinant vaccine platform is a leading candidate for prevention of hemorrhagic fevers in West Africa. The successful results of the VSV-Ebola (VSVΔG/EBOVGPC) ring vaccine trials during the 2014/2015 Ebola virus outbreak underscore the potential of the vaccine as a preventative measure for hemorrhagic fevers.^7^ This and other ongoing safety trials point to a safe and immunogenic vaccine platform with over 20,000 participants receiving the VSVΔG/EBOVGPC vaccine to date. Using the same vaccine platform, a VSV-based LASV vaccine (VSVΔG/LASVGPC) was developed in parallel.^8^ The current VSVΔG/LASVGPC vaccine candidate was originally designed with the glycoprotein (GPC) isolated from a Clade IV LASV (Josiah). LASV contains significant genetic heterogeneity with as many as seven clades spread across much of West Africa, making the development of a universal vaccine difficult.^9^ Additionally, very few studies have measured the long-term immunity and efficacy of the VSV platform, while none have specifically addressed the VSVΔG/LASVGPC vaccine.^10^ While trials to examine the safety, immunogenicity, and efficacy of the VSVΔG/LASVGPC vaccine would be needed, the recent EBOV vaccine trials suggest that the VSV platform will be safe and widely accepted for the prevention of hemorrhagic fever in West Africa.

Nigeria has suffered an unprecedented resurgence of LASV from 2015 to 2018. As early as April 2018, 21 states had at least one confirmed case of rodent-borne transmission. With new infections on the decline the critical phase of the outbreak is now under control. Suspected cases reached 2623 this year alone with 514 confirmed positive and 134 deaths leading to a case fatality rate of 26.1%. Additionally, 39 health care workers have been infected with eight deaths.^11^ Coupled with high disease burden in West Africa, the potential for person–person transmission, and imported cases, makes LF a significant health priority for research and development.

Historically there have been two animal models described for studying LF; inbred (strain 13) guinea pigs (GPs), and non-human primates (NHPs).^12^ More recently, a guinea pig-adapted (GPA) LASV model of infection has been characterized for testing vaccines and therapeutics.^13,14^ In this study, outbred Hartley guinea pigs were vaccinated with a single dose of VSVΔG/LASVGPC and challenged as early as 7 days or as late as 1 year after vaccination with a lethal dose of GPA-LASV; Josiah. Rapid and long-term protection from LASV challenge was achieved with sustained immunogenicity over a 1-year time span. These data indicate that the VSVΔG/LASVGPC vaccine could provide rapid and sustained long-term prevention against LASV disease and mortality in West Africa.

## Results

### Rapid protection from lethal LASV challenge with a single dose of VSVΔG-LASVGPC vaccine

**Fig. 1:**
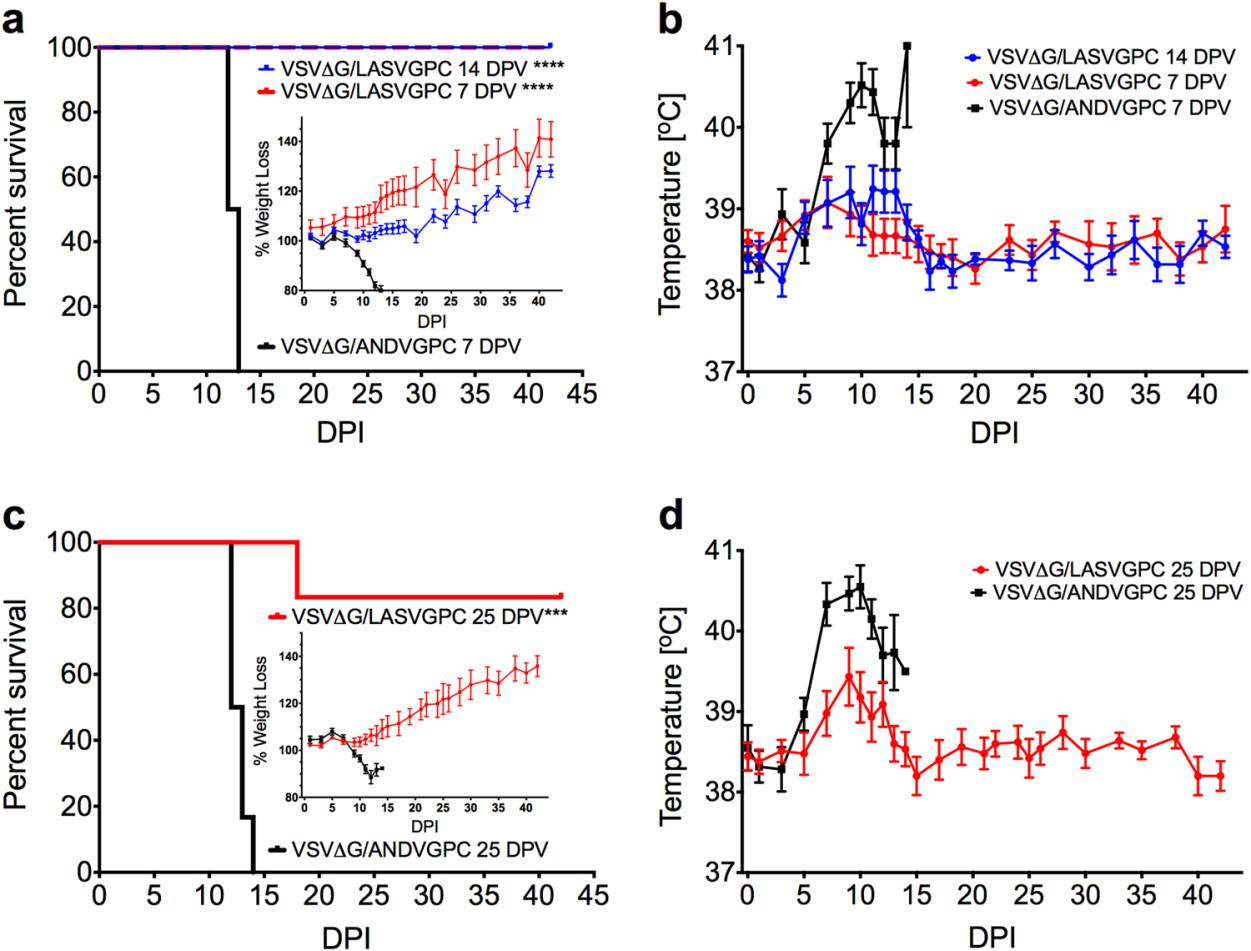
Rapid protection from lethal Lassa virus challenge with a single dose of VSVΔG-LASVGPC vaccine. **a** Survival, weight loss (inset) and **b** temperature analysis of Hartley guinea pigs challenged 14 and 7 days post-vaccination (*n* = 6). **c** Survival, weight loss (inset) and **d** temperature analysis of Harley guinea pigs challenged 25 days post-vaccination (*n* = 6). Mantel-Cox survival analysis was used and all data are presented as mean values with error bars indicating SEM. *p* = 0.1234 (ns), 0.0332 (*), 0.0021 (**), 0.0002 (***), <0.0001 (****)

**Fig. 2:**
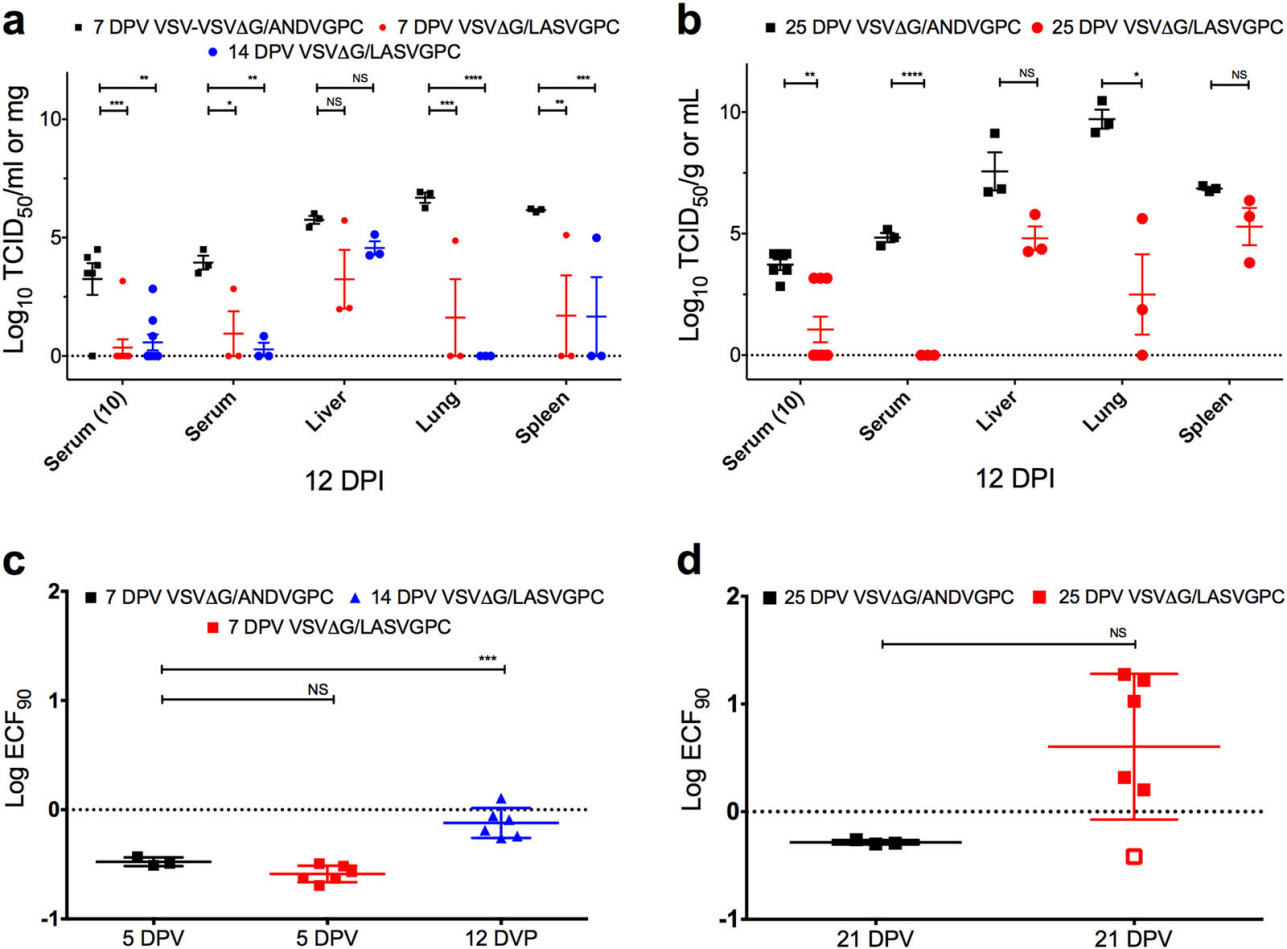
Viral burden and serological analysis of rapid vaccination with VSVΔG-LASVGPC. TCID_50_ analysis of serum, liver, lung and spleen collected from animals vaccinated **a** 7/14 DPV (2-Way Anova; Dunnett’s) and **b** 25 DPV (*T*-test; Holm-Sidak). Total LASVGPC-specific IgG titers were assessed by endpoint titration and the Log ECF_90_ for each time-point calculated for animals challenged **c** 7/14 DPV (One-Way Anova; Dunnett’s) and **d** 25 DPV (Two-tailed *T*-test), respectively. Data are presented as mean values with error bars indicating SEM. *p* = 0.1234 (ns), 0.0332 (*), 0.0021 (**), 0.0002 (***), <0.0001 (****)

In order to evaluate the ability of VSVΔG-LASVGPC to rapidly protect outbred Hartley guinea pigs from lethal GPA-LASV, we vaccinated groups of nine female guinea pigs (4–5 weeks old) with 1 × 10^6^ PFU of VSVΔG-LASVGPC by the intraperitoneal route. An additional 6 control animals were vaccinated with an analogous vaccine containing Andes virus GPC (VSVΔG-ANDVGPC) as vector control. On 7 and 14 days post-vaccination (DPV) all guinea pigs were challenged with a lethal dose of GPA-LASV (1 × 10^4^ TCID_50_ /10x LD_50_, i.p) and monitored for signs of disease for up to 42 days (Fig. 1a). All animals receiving the VSVΔG-LASVGPC vaccine survived infection with no apparent signs of disease, and steadily gained weight throughout the experiment. The 7 and 14 DPV animals achieved 100% protection (*p* < 0.0002, Mantel-Cox) compared to control animals (VSVΔG-ANDVGPC). Both VSVΔG-LASVGPC groups had slightly elevated temperatures as monitored by temperature transponder with an average of 39.2 °C from days 10–14 before dropping to regular baseline measurement for the remainder of the experiment (Fig. 1b). Control animals exhibited signs of disease characterized by significant weight loss beginning on day 8, reaching >20% by day 14 (Fig. 1a, inset), which was accompanied by high fever (average 40.5; 10 days post-infection), before advancing to respiratory distress and hypothermia, at which time the humane endpoints were achieved resulting in animal euthanasia. A group of three random VSVΔG-LASVGPC vaccinated animals were euthanized on day 12 post-infection when control animals began to reach the humane endpoint in order to collect tissues and blood for comparison. In addition, serum was collected from all animals 10 days post-infection in order to assess viremia (Fig. 2a). The 7 and 14 DPV VSVΔG-LASVGPC animals had significantly less infectious virus in the serum compared to vector control animals on days 10 and 12 post-infection. These animals also had significantly less infectious virus in the lung (mean difference of 5 and 6.7 Log TCID_50_/ml-mg, respectively) and spleen (mean difference of 4.5 and 4.5 Log TCID_50_/ml-mg, respectively) as compared to control animals (Fig. 2a). While the samples collected from the liver had less infectious virus compared to controls it did not reach statistical significance (Supplementary Table 1). Despite the VSVΔG-LASVGPC vaccine providing 100% protection from lethal disease, infectious virus was detectable in the serum, liver, lung and spleen of some animals (7 and 14 DPV) indicating that sterilizing immunity was not uniformly achieved. Total LASVGPC-specific IgG titers were assessed by endpoint dilution and the log dilution at which 90% of the sample bound LASVGPC was calculated. We found no significant difference between serum titers 5 days post-vaccination between VSVΔG-LASVGPC animals and VSVΔG-ANDVGPC controls (Fig. 2c). However despite extremely low LASVGPC-specific IgG titers we were able to detect a significant difference in titers 12 days post-vaccination (*p* < 0.0001, One-way Anova; Dunnett’s).

A second set of animals was again vaccinated with VSVΔG-LASVGPC and then challenged 25 DPV with a lethal dose of GPA-LASV in order to test the durability of protection after approximately one month. Control animals began to rapidly lose weight on day 9 post-infection (Fig. 1c, inset) which was also preceded by a rapid increase in average basal temperature beginning on day 5 post-infection. The control animals sustained a high fever from day 7 onwards reaching a peak of 40.5 °C. A rapid drop in temperature (hypothermia) coinciding with significant weight loss (>20%) and respiratory distress resulted in humane euthanasia. In contrast, VSVΔG-LASVGPC vaccinated animals steadily gained weight throughout the experiment, and generally showed no physical signs of disease with the exception of a low-grade fever reaching a mean peak of 39.4 °C on day 9 post-infection (Fig. 1d). The 25 DPV guinea pigs achieved statistically significant protection of 83.3% with one VSVΔG-LASVGPC animal succumbing to disease (Fig. 1c, *p* = 0.0005, Mantel-Cox). This single animal had delayed disease on-set compared to controls with a high-grade fever reaching a peak of 40.6 °C by day 12 post-infection and mild weight loss (8%). At 18 days post-infection the animal became hypothermic and showed signs of respiratory distress requiring euthanasia. Interestingly, this animal had no LASVGP-specific IgG antibody on day 21 similar to VSVΔG-ANDVGPC controls (Fig. 2d), which could suggest a failed immunization. A group of three VSVΔG-LASVGPC vaccinated animals were euthanized; serum and tissue were collected for comparison with control animals on day 12 post-infection in addition to all animals being sampled on day 10 post-infection (Fig. 2b). Control animals had infectious virus titers that were 4.8 Log TCID_50_/ml higher in the serum compared to VSVΔG-LASVGPC vaccinated animals on day 10 (*p* < 0.0001, Two-tailed *T*-test; Holm-Sidak). As before, some VSVΔG-LASVGPC animals had detectable viremia on day 10 (Fig. 2b). Control animals also had significantly higher titers of virus in the lung compared to VSVΔG-LASVGPC animals with a mean difference of 7.2 Log TCID_50_/mg (*p* = 0.0389, Two-tailed *T*-test; Holm-Sidak). Twenty-one days post-vaccination VSVΔG-LASVGPC vaccinated animals reached an average peak antibody titer of 0.47 Log ECF_90_ that was not significantly different than the VSVΔG-ANDVGPC controls due to the large range/variance in values (Fig. 2d).

### Long-term protection from lethal LASV challenge with a single dose of VSVΔG-LASVGPC vaccine

**Fig. 3:**
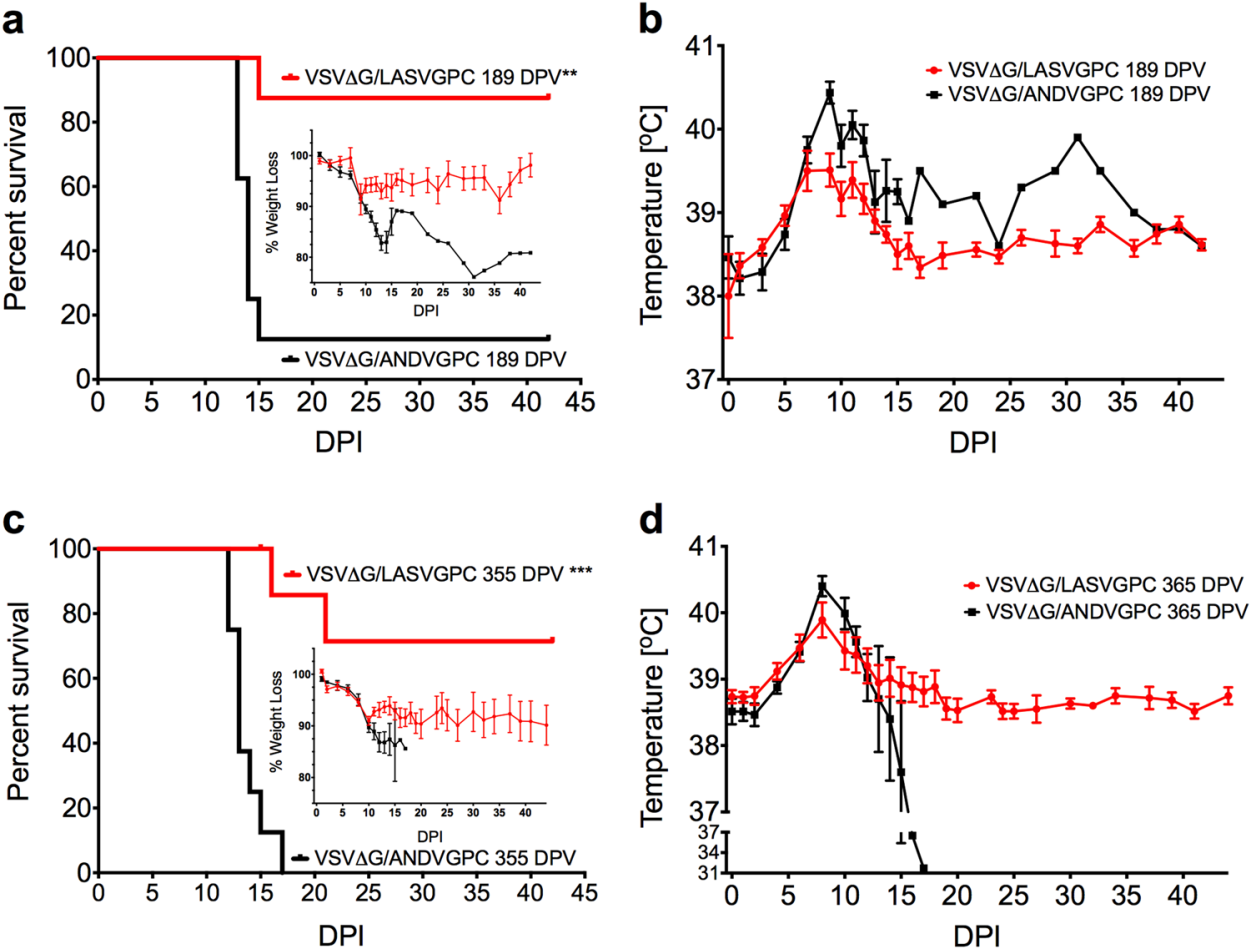
Long-term protection from lethal Lassa virus challenge with a single dose of VSVΔG-LASVGPC vaccine. **a** Survival, weight loss (inset) and **b** temperature analysis of Hartley guinea pigs challenged 189 days post-vaccination (*n* = 8). **c** Survival, weight loss (inset) and **d** temperature analysis of Harley guinea pigs challenged 355 days post-vaccination (*n* = 8). Mantel-Cox survival analysis was used and data are presented as mean values with error bars indicating SEM. *p* = 0.1234 (ns), 0.0332 (*), 0.0021 (**), 0.0002 (***), <0.0001 (****)

**Fig. 4:**
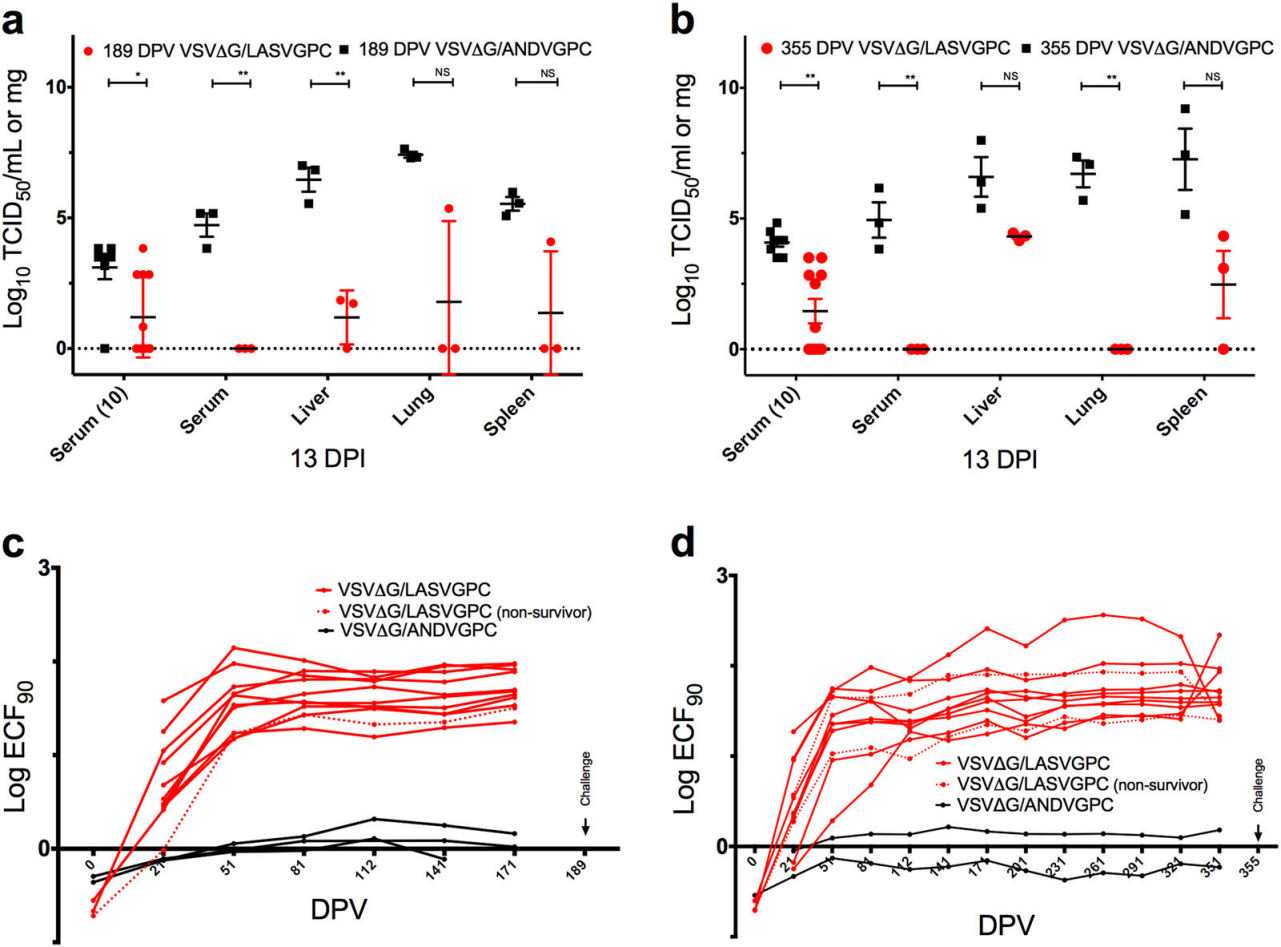
Viral burden and serological analysis of long-term vaccination with VSVΔG-LASVGPC. TCID_50_ analysis of serum, liver, lung and spleen collected from animals vaccinated **a** 189 DPV and **b** 355 DPV (*T*-test; Holm-Sidak). Total LASVGPC-specific IgG titers were assessed by endpoint titration and the Log ECF_90_ for each time-point calculated for animals challenged **c** 189 DPV and **d** 355 DPV, respectively. Data are presented as mean values with error bars indicating SEM. *p* = 0.1234 (ns), 0.0332 (*), 0.0021 (**), 0.0002 (***), <0.0001 (****)

Long-term immunogenicity and protection studies with respect to the VSV vaccine platform in animal models are rare,^10,15,16^ owing to the extensive husbandry requirements, with none specifically studying the longevity of the VSVΔG-LASVGPC vaccine. With this in mind we vaccinated groups of 11 guinea pigs with 1 × 10^6^ PFU of VSVΔG-LASVGPC as well as an accompanying vector control vaccine (VSVΔG-ANDVGPC). Guinea pigs were challenged 6 months (189 DPV) after vaccination with a lethal dose of GPA-LASV (1 × 10^4^ TCID_50_, i.p) and monitored for signs of disease for up to 42 days (Fig. 3a,b). Of the eight animals vaccinated with VSVΔG-LASVGPC and challenged 6 months later, seven guinea pigs survived, leading to a statistically significant protection of 87.5% (*p* = 0.0014, Mantel-Cox). Guinea pigs that were challenged 6 months after vaccination with VSVΔG-LASVGPC lost on average 10% of their starting weight by day 10 post-infection and then recovered by the end of the experiment (Fig. 3a, inset). Additionally, VSVΔG-LASVGPC vaccinated animals maintained a low-grade fever from day 5–13 post-infection with a mean peak temperature of 39.3 °C on day 11 (Fig. 3b). VSVΔG-ANDVGPC control animals initially followed the same disease course, however they continued to lose weight 10 days post-infection, which was accompanied by a peak fever of 40 °C on day 11. These animals also developed hypothermia and respiratory distress requiring humane euthanasia. A single animal vaccinated with the VSVΔG-ANDVGPC control vaccine survived lethal challenge despite seroconverting with a high IgG LASV-NP titer (Supplementary Fig. 1). In order to assess tissue related virus burden, three VSVΔG-LASVGPC vaccinated animals were euthanized on day 13 post-infection at a time when the VSVΔG-ANDVGPC controls were succumbing to disease. In all cases VSVΔG-LASVGPC vaccinated animals had less infectious virus in the serum, liver, lung and spleen compared to controls (Fig. 4a). In particular, VSVΔG-LASVGPC vaccinated animals had a mean difference of 4.7 and 5.3 Log TCID_50_/ml-mg isolated from the serum and liver on day 12 post-infection respectively, compared to VSVΔG-ANDVGPC control animals.

With the need to test the long-term immunity generated by the VSVΔG-LASVGPC vaccine we housed guinea pigs for up to one year (355 days) after a single vaccination. Animals were challenged with a lethal dose of GPA-LASV and monitored for signs of disease (Fig. 3c, d). Two out of nine VSVΔG-LASVGPC vaccinated animals succumbed to infection with delayed disease onset, 16 and 21 days post-infection, respectively. A single dose of VSVΔG-LASVGPC protected 71% of guinea pigs 1 year after vaccination from lethal LASV challenge (*p* = 0.0001, Mantel-Cox). On average the VSVΔG-LASVGPC vaccinated animals lost 10% of their starting weight by day 12, similar to those in the 6-month challenge group. Additionally they reached an average peak temperature of 39.9 °C while the VSVΔG-ANDVGPC controls reached an average peak temperature of 40.4 °C by day 8 post-infection. As before with the previous experiment some but not all VSVΔG-LASVGPC vaccinated animals had detectable infectious virus in the serum on day 10 post-infection with a mean difference of 2.6 Log TCID_50_/ml compared to VSVΔG-ANDVGPC controls (*p* = 0.001, Two-tailed *T*-test; Holm-Sidak) (Fig. 4b). Tissues collected from 3 guinea pigs on day 13 post-infection also had less virus compared to controls in the liver, lung and spleen with a mean difference of 2.3, 6.7, and 4.8 Log TCID_50_/mg, respectively (Fig. 4b).

Total IgG LASVGPC-specific antibody titers were assessed by end point dilution every month for 6 months and 1 year following vaccination (Fig. 4c, d). Almost all VSVΔG-LASVGPC vaccinated animals reached peak IgG titers by day 51 which were sustained up until the final sampling date (171 or 351 DPV). However, the few animals that did succumb to disease had delayed antibody responses compared to the majority of VSVΔG-LASVGPC vaccinated animals on day 21 DPV while survivors generally had higher VSV-LASVGPC IgG responses (Fig. 4c, d; dashed line).

## Discussion

There are numerous vaccine platforms that have shown protection in animal models against LASV infection, with one of the leading candidates being identified as the VSVΔG-LASVGPC vaccine.^2–5,14,17–23^ The genetic heterogeneity associated with LASV poses a significant challenge to the development of a universal vaccine to protect across a wide geographical region and multiple genotypes. However, testing of the VSVΔG-LASVGPC vaccine, which was originally designed to protect against LASV-Josiah (a Sierra Leone isolate) has now been shown to protect against several genetically and geographically distinct isolates.^2,5,6,24^ With this in mind as well as the lack of short and long-term protection studies in the literature for the VSVΔG-LASVGPC (Josiah) vaccine, we tested the ability of the vaccine to protect outbred Hartley guinea pigs from lethal LASV challenge between one week and one year following a single vaccination.

Guinea pigs challenged 7 and 14 days after VSVΔG-LASVGPC vaccination were afforded 100% protection from lethal LASV infection. While animals challenged 25 days after vaccination indicated 83.3% protection. Often, as has been shown in strain 13 guinea pigs, VSVΔG-LASVGPC vaccination results in sterilizing immunity with no signs of clinical disease.^7,24^ While the protective efficacy of the vaccine remains relatively unchanged between the Strain 13 and Hartley guinea pig models, some VSVΔG-LASVGPC vaccinated Hartley guinea pigs did show signs of disease including weight loss, low-grade fever, and detectable viremia 10 days post-infection. The VSVΔG-LASVGPC vaccine protective efficacy, viremia, and serology align more closely with protective data generated from NHP studies (a model that more accurately reproduces human pathogenicity).^8,25^ These short-term studies suggest that the GPA-LASV virus generated for testing in the outbred guinea pig model may be a more robust model of protection as compared to strain 13 guinea pigs for vaccine and therapeutic testing. A dose of 1 × 10^6^ PFU was used for vaccination of all animals in our study while human and NHP studies have often used much higher doses (≥1 × 10^7^ PFU). This discrepancy in vaccination dose may lead to sterilizing immunity or higher levels of long-term protection if doses of ≥1 × 10^7^ PFU are used in the future. It is worth noting that all the control animals receiving VSVΔG-ANDVGPC 7 days before lethal challenge succumbed to infection, indicating a LASV-specific short term response, ruling out innate stimulation from the vector itself as seen with previous VSV-vaccine studies.^9,26^

When testing the long-term immunogenicity and protection of a single VSVΔG-LASVGPC vaccination, 87.5 and 71% protection at 6 months (189 days) and 1 year (355 days) was achieved after a lethal LASV challenge, respectively. VSVΔG-LASVGPC vaccinated animals that succumbed to infection had delayed time to peak antibody titers. (Fig. 4c, d; dashed lines). Interestingly on day 21 post-vaccination these animals had similar LASVGPC-specific antibody titers to the VSVΔG-ANDVGPC vaccinated animals before eventually reaching peak titers by 51 days post-vaccination. The two non-surviving animals that were challenged 1 year after infection (Fig. 4d; dashed lines) had the lowest peak LASVGPC-specific IgG titers at the time of challenge in the VSVΔG-LASVGPC vaccinated group. Analysis of LASVGPC-specific IgG in survivors and non-survivors just prior to challenge, 189 and 355 post-vaccination, revealed a significant correlation between total LASVGPC-specific IgG and survival (Supplementary Fig. 2; *p* = 0.0334, Two-tailed *T*-test). This is in line with a recent publication identifying non-neutralizing LASVGPC-specific antibodies elicited by a LASV-Rabies vaccine as a possible correlate of protection.^27^ However, given that there were only 3 non-survivors for comparison in our study a larger study is warranted to evaluate if antibody titers generated by VSVΔG-LASVGPC at the time of challenge correlate with survival. Recently NHP studies have shown that human neutralizing antibodies administered as late as 8 days post-infection can protect from lethal LASV infection.^10,28^ With this in mind we cannot rule out a role for cell-mediated protection during infection. Development of vaccines that include T-cell epitopes identified in NP or L would also be of considerable interest. Unfortunately, reagents to measure cell-mediated immune responses in the guinea pig model are lacking and limit our ability to fully assess the immunological correlates of protection in this model.

Our data suggests that the outbred Hartley guinea pig model for LASV infection could be a robust model for vaccine and therapeutic testing. While the guinea pig model for LASV infection has slightly different pathogenesis characteristics than human LF disease, protective efficacy and viremia parameters correlate closely with previous VSVΔG-LASVGPC testing in NHP models of disease.^12,25^ Efforts should be made to adapt other geographically and genetically distinct isolates for testing of vaccine candidates in the Hartley guinea pig model. We have also shown for the first time that the VSVΔG-LASVGPC vaccine can provide rapid and durable long-term protection from lethal LASV infection. Further, studies are warranted in NHPs to discover the long-term potential of the VSVΔG-LASVGPC vaccine as a universal candidate for LF prevention in West Africa.

## Methods

### Ethics

The Public Health Agency of Canada Animal Care Committee approved all animal work for this study. Certified personnel, adhering to the guidelines set forth by the Canadian Council on Animal Care (CCAC), conducted all animal procedures. All animals reaching a predetermined endpoint score were humanely euthanized in accordance with CCAC guidelines.

### Viruses

Lethal infection of Hartley guinea pigs was achieved by passaging LASV Josiah in Hartley guinea pigs four times (GPA-LASV) until a uniformly lethal phenotype was achieved.^13,14^ The virus was propagated in VeroE6 (ATCC, CRL-1586) cells and titered using the Spearman-Karber standard 50% tissue culture infectious dose (TCID_50_) method.

### Guinea pigs

Four-week-old outbred female Hartley guinea pigs (Charles River, Canada) were vaccinated and housed for up to a year in groups of 6–9 animals in Biosafety Level 2 (BSL2) animal facilities before being transferred to the BSL4 environment. A minimum of one-week acclimation was observed before the start of any experimental procedures. Animals were implanted subcutaneously just prior to transfer to BSL4 with an electronic transponder temperature chip (Bio Medic Data Systems Inc.) in order to identify and monitor the animals throughout the experiment. Guinea pigs were housed in caging units that provide negative pressure and HEPA-filtered containment in the National Microbiology Laboratory BSL4 for LASV challenge.

### In-vivo efficacy studies

All guinea pigs were vaccinated with either a single dose of recombinant VSVΔG-ANDVGPC as a control or VSVΔG-LASVGPC (1 × 10^6^ PFU) by the intraperitoneal route. Guinea pigs were housed for 7 (9/group), 14 (9/group), 25 (9/group), 189 (11/group), and 355 (11/group) days post-vaccination. Guinea pigs were sampled 1 week, 3 weeks, and every month thereafter following vaccination in order to assess total LASVGP-specific IgG titers. On the respective time points, animals were transferred to the BSL4 facility and challenged with a lethal dose of guinea pig-adapted LASV (1 × 10^4^ TCID_50_/10× LD_50_) by the intraperitoneal route. Animals were weighed and temperatures were taken on a daily basis in addition to daily clinical scoring. At a time when control (VSVΔG-ANDVGPC immunized GPs) animals experienced advanced signs of disease requiring euthanasia, 3 random guinea pigs from the VSVΔG-LASVGPC vaccinated groups were euthanized to compare viral titers, and tissue specific replication. The remaining guinea pigs were monitored for survival until day 42 post-infection or until endpoint scoring was achieved requiring euthanasia.

### TCID_50_ determination

VeroE6 cells were seeded into 96-well tissue culture plates in order to become 70–80% confluent within 24 h. Tissues were homogenized in 1 ml of DMEM using a TissueLyserII (Qiagen). The homogenates were clarified by centrifugation at 10,000×*g* for 10 min. Clarified homogenates or serum were ten-fold serial diluted in DMEM containing 2% FBS and 100 μl of each dilution in triplicate were added per well. Cells were then incubated for 7 days at 37 °C with 5% CO_2_. Cells were then assessed for virus-induced cytopathic effect and the tissue culture infectious dose (TCID_50_) was calculated by the Spearman-Karber method.^29^

### Lassa virus serology and seroconversion

In order to measure the total IgG LASVGPC-specific immune responses serum was collected once a month from all vaccinated animals. Additionally to ensure surviving guinea pigs received an adequate challenge, serum samples were collected from all surviving animals at day 42 post-infection. A commercial ReLASV^TM^ Pan-Lassa GP or NP IgG/IgM ELISA (Zalgen Labs) was modified to measure total guinea pig-specific IgG to LASV GP or NP as follows. Briefly, guinea pig serum samples were diluted 1 in 100 in order to measure NP specific responses and incubated for 30 min at room temperature on pre-coated ELISA plates with recombinant nucleoprotein. Plates were washed four times with the kit provided wash buffer. A secondary goat anti-guinea pig specific IgG (H&L)-HRP (SeraCare) antibody replaced the kit provided antibody and was diluted at 1:1000. Plates were again incubated for 30 min at room temperature followed by 4 washes. The kit provided one-component substrate was added to the plates and incubated for 10 min. The reaction was stopped using 0.36 N sulfuric acid and the OD of samples read at 405 nm.

### Statistical analysis

Statistical analyses were conducted using GraphPad Prism 7.0. A log rank Mantel-Cox test was used for survival analysis. Infectious virus comparisons in the serum and tissues were performed with either a 2-way Anova or multiple *t*-tests corrected for multiple comparisons by the Holm-Sidak method. *p* values of <0.05 were considered significant.

### Reporting summary

Further information on research design is available in the Nature Research Reporting Summary linked to this article.

## Supplementary information

Supplemental Material

Life Sci Reporting Summary

## Data Availability

The authors declare that all data supporting the findings of this study are available within the paper and supplementary files.

## Supplementary information

**Supplementary information** accompanies the paper on the *npj Vaccines* website (10.1038/s41541-019-0104-x).

## References

[1] Monath TP, Newhouse VF, Kemp GE, Setzer HW, Cacciapuoti A (1974). Lassa virus isolation from mastomys natalensis rodents during an epidemic in Sierra Leone. Science.

[2] McCormick JB (1987). A case–control study of the clinical diagnosis and course of lassa fever. J. Infect. Dis..

[3] Frame JD (1989). Clinical features of lassa fever in liberia. Rev. Infect. Dis..

[4] Bausch DG (2001). Lassa fever in guinea: I. Epidemiology of human disease and clinical observations. Vector-Borne Zoonotic Dis..

[5] Ogbu, O., Ajuluchukwu, E. & Uneke, C. J. Lassa fever in West African sub-region: an overview. *J Vector Borne* **44**, 1–11 (2007). 17378212

[6] Fisher-Hoch, S. P., Tomori, O. & Nasidi, A. Review of cases of nosocomial Lassa fever in Nigeria: the high price of poor medical practice. *BMJ* **311**, 857–859 (1995). 10.1136/bmj.311.7009.857 PMC2550858 7580496

[7] Henao-Restrepo AM (2015). Efficacy and effectiveness of an rVSV-vectored vaccine expressing Ebola surface glycoprotein: interim results from the Guinea ring vaccination cluster-randomised trial. Lancet.

[8] Garbutt M (2004). Properties of replication-competent vesicular stomatitis virus vectors expressing glycoproteins of filoviruses and arenaviruses. J. Virol..

[9] Warner BM, Safronetz D, Stein DR (2018). Current research for a vaccine against Lassa hemorrhagic fever virus. DDDT.

[10] Wong G (2014). Immunization with vesicular stomatitis virus vaccine expressing the Ebola glycoprotein provides sustained long-term protection in rodents. Vaccine.

[11] An update of Lassa fever outbreak in Nigeria. *NCDC* 1–4 (2018).

[12] Gowen BB, Holbrook MR (2008). Animal models of highly pathogenic RNA viral infections: hemorrhagic fever viruses. Antivir. Res..

[13] Safronetz D (2015). The broad-spectrum antiviral favipiravir protects guinea pigs from lethal Lassa virus infection post-disease onset. Sci. Rep..

[14] Cross RW (2016). Treatment of Lassa virus infection in outbred guinea pigs with first-in-class human monoclonal antibodies. Antivir. Res..

[15] Mire CE (2014). Durability of a vesicular stomatitis virus-based marburg virus vaccine in nonhuman primates. PLoS One.

[16] Fausther-Bovendo, H., Alimonti, J. B. & Kobinger, G. P. Immunization with vesicular stomatitis virus vaccine expressing the Ebola glycoprotein provides sustained long-term protection in rodents. *Vaccine* **32**, 5722–5729(2014). 10.1016/j.vaccine.2014.08.028 PMC7115511 25173474

[17] Pushko P, Geisbert J, Parker M, Jahrling P, Smith J (2001). Individual and bivalent vaccines based on alphavirus replicons protect guinea pigs against infection with Lassa and Ebola viruses. J. Virol..

[18] Wang M, Jokinen J, Tretyakova I, Pushko P, Lukashevich IS (2018). Alphavirus vector-based replicon particles expressing multivalent cross-protective Lassa virus glycoproteins. Vaccine.

[19] McCormick JB, Mitchell SW, Kiley MP, Ruo S, Fisher-Hoch SP (1992). Inactivated Lassa virus elicits a non protective immune response in rhesus monkeys. J. Med. Virol..

[20] Cashman KA (2013). Enhanced efficacy of a codon-optimized DNA vaccine encoding the glycoprotein precursor gene of Lassa virus in a Guinea Pig Disease Model when delivered by dermal electroporation. Vaccines.

[21] Cashman KA (2017). DNA vaccines elicit durable protective immunity against individual or simultaneous infections with Lassa and Ebola viruses in guinea pigs. Hum. Vaccin Immunother..

[22] Lukashevich IS, Vasiuchkov AD, Stel’makh TA, Scheslenok EP, Shabanov AG (1991). The isolation and characteristics of reassortants between the Lassa and Mopeia arenaviruses. Vopr. Virusol..

[23] Lukashevich IS (2008). Safety, immunogenicity, and efficacy of the ML29 reassortant vaccine for Lassa fever in small non-human primates. Vaccine.

[24] Safronetz D (2015). A recombinant vesicular stomatitis virus-based Lassa fever vaccine protects guinea pigs and macaques against challenge with geographically and genetically distinct Lassa viruses. PLoS Negl. Trop. Dis..

[25] Geisbert TW (2005). Development of a new vaccine for the prevention of Lassa fever. PLoS Med..

[26] Brown KS, Safronetz D, Marzi A, Ebihara H, Feldmann H (2011). Vesicular stomatitis virus-based vaccine protects hamsters against lethal challenge with Andes virus. J. Virol..

[27] Abreu-Mota T (2018). Non-neutralizing antibodies elicited by recombinant Lassa-Rabies vaccine are critical for protection against Lassa fever. Nat. Commun..

[28] Mire CE (2017). Human-monoclonal-antibody therapy protects nonhuman primates against advanced Lassa fever. Nat. Med..

[29] Ramakrishnan MA (2016). Determination of 50% endpoint titer using a simple formula. World J. Virol..

