# Supplementary material for: A recombinant vesicular stomatitis-based Lassa fever vaccine elicits rapid and long-term protection from lethal Lassa virus infection in guinea pigs
Source: NPJ Vaccines. 2019 Feb 8;4:8. doi: 10.1038/s41541-019-0104-x (PMC6368541; doi:10.1038/s41541-019-0104-x)
Supplement: Supplementary file 1 — Supplemental Material [file 41541_2019_104_MOESM1_ESM.pdf]

Figure	Test	Sample	Comparison	Mean Diff.	Significant	Summary	Adj.P Value	Alpha
2A	Dunnett's	Serum (10)	7 DPV VSV-ANDVGP vs. 14 DPV VSV-LASVGP	2.676	Yes	**		0.05
2A	Dunnett's	Serum (10)	7 DPV VSV-ANDVGP vs. 7 DPV VSV-LASVGP	2.898	Yes	***		0.05
2A	Dunnett's	Serum	7 DPV VSV-ANDVGP vs. 14 DPV VSV-LASVGP	3.666	Yes	**		0.05
2A	Dunnett's	Serum	7 DPV VSV-ANDVGP vs. 7 DPV VSV-LASVGP	3	Yes	*		0.05
2A	Dunnett's	Liver	7 DPV VSV-ANDVGP vs. 14 DPV VSV-LASVGP	1.187	No	ns		0.05
2A	Dunnett's	Liver	7 DPV VSV-ANDVGP vs. 7 DPV VSV-LASVGP	2.509	No	ns		0.05
2A	Dunnett's	Lung	7 DPV VSV-ANDVGP vs. 14 DPV VSV-LASVGP	6.686	Yes	****		0.05
2A	Dunnett's	Lung	7 DPV VSV-ANDVGP vs. 7 DPV VSV-LASVGP	5.062	Yes	***		0.05
2A	Dunnett's	Spleen	7 DPV VSV-ANDVGP vs. 14 DPV VSV-LASVGP	4.498	Yes	***		0.05
2A	Dunnett's	Spleen	7 DPV VSV-ANDVGP vs. 7 DPV VSV-LASVGP	4.46	Yes	**		0.05
2B	Holm-Sidak	Serum (10)	25 DPV VSV-ANDVGP vs. 25 DPV VSV-LASVGP	2.667	Yes	**	0.0069	0.05
2B	Holm-Sidak	Serum	25 DPV VSV-ANDVGP vs. 25 DPV VSV-LASVGP	4.833	Yes	****	<0.0001	0.05
2B	Holm-Sidak	Liver	25 DPV VSV-ANDVGP vs. 25 DPV VSV-LASVGP	2.755	No	ns	0.08	0.05
2B	Holm-Sidak	Lung	25 DPV VSV-ANDVGP vs. 25 DPV VSV-LASVGP	7.213	Yes	*	0.0389	0.05
2B	Holm-Sidak	Spleen	25 DPV VSV-ANDVGP vs. 25 DPV VSV-LASVGP	1.565	No	ns	0.1117	0.05
4A	Holm-Sidak	Serum (10)	189 DPV VSV-ANDVGP vs. 189 DPV VSV-LASVGP	1.907	Yes	*	0.0322	0.05
4A	Holm-Sidak	Serum	189 DPV VSV-ANDVGP vs. 189 DPV VSV-LASVGP	4.723	Yes	**	0.0022	0.05
4A	Holm-Sidak	Liver	189 DPV VSV-ANDVGP vs. 189 DPV VSV-LASVGP	5.271	Yes	**	0.0088	0.05
4A	Holm-Sidak	Lung	189 DPV VSV-ANDVGP vs. 189 DPV VSV-LASVGP	5.63	No	ns	0.068	0.05
4A	Holm-Sidak	Spleen	189 DPV VSV-ANDVGP vs. 189 DPV VSV-LASVGP	4.177	No	ns	0.068	0.05
4B	Holm-Sidak	Serum (10)	355 DPV VSV-ANDVGP vs. 355 DPV VSV-LASVGP	2.629	Yes	**	0.001	0.05
4B	Holm-Sidak	Serum	355 DPV VSV-ANDVGP vs. 355 DPV VSV-LASVGP	4.945	Yes	**	0.0056	0.05
4B	Holm-Sidak	Liver	355 DPV VSV-ANDVGP vs. 355 DPV VSV-LASVGP	2.281	No	ns	0.0785	0.05
4B	Holm-Sidak	Lung	355 DPV VSV-ANDVGP vs. 355 DPV VSV-LASVGP	6.707	Yes	**	0.001	0.05
4B	Holm-Sidak	Spleen	355 DPV VSV-ANDVGP vs. 355 DPV VSV-LASVGP	4.792	No	ns	0.0785	0.05

0.1234 (ns), 0.0332 (*), 0.0021 (**), 0.0002 (***), <0.0001 (****)

Supplemental Table 1. Statistical summary of viremia comparisons from serum and tissues collected during short and long-term VSVΔG-LASVGPC vaccination experiments.

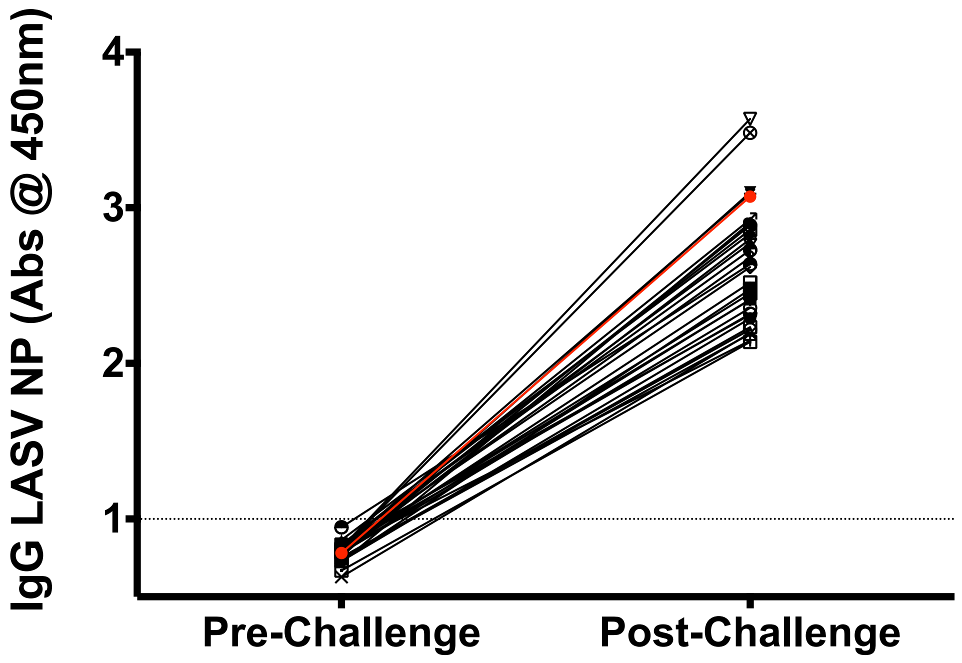

Supplemental Figure 1. LASV seroconversion of all surviving guinea pigs challenged with GPA-LASV. A single VSV-ANDV vaccinated control animal survived GPA-LASV challenge (Figure 3A), however seroconversion was confirmed indicating successful LASV infection (red).

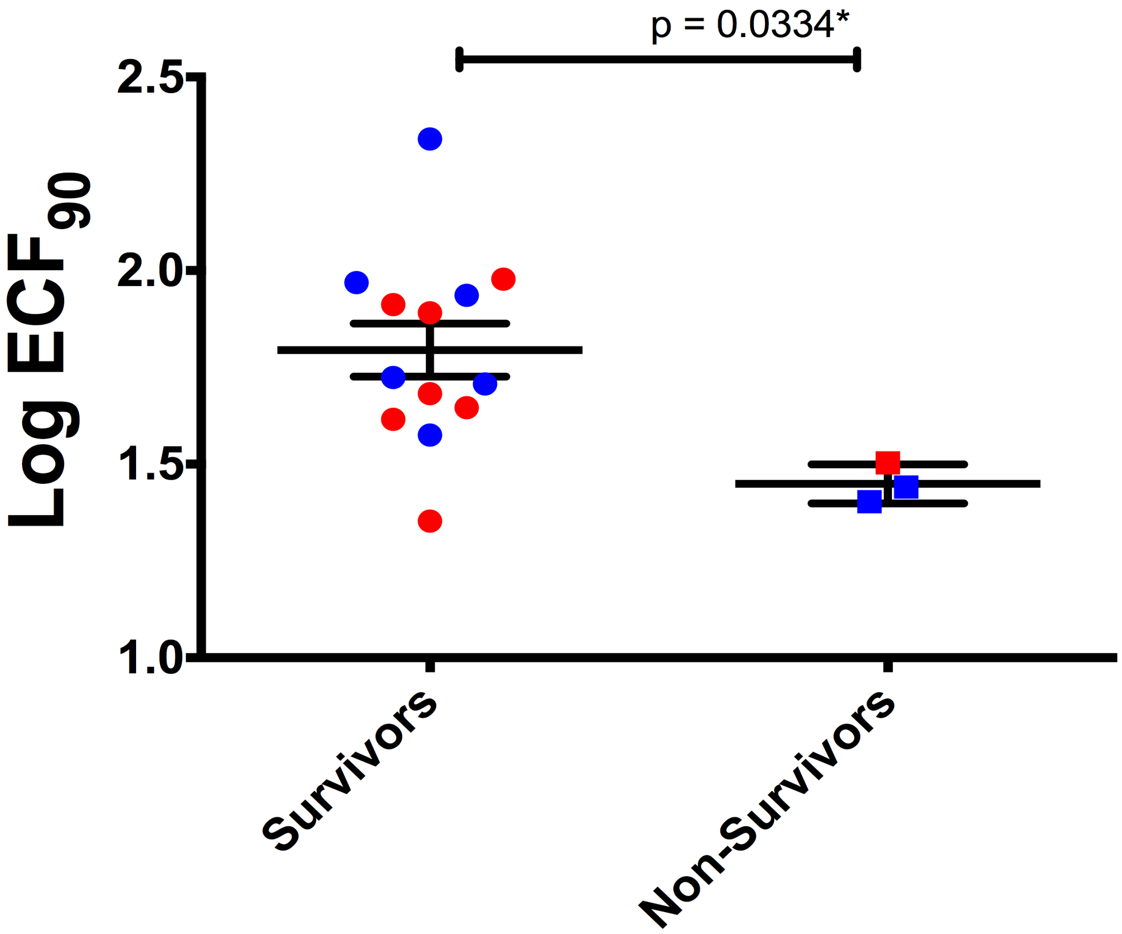

Supplemental Figure 2. Total LASVGPC-specific IgG measured by endpoint dilution in survivors compared to non-survivors challenged 189 (red) and 355 (blue) days post-vaccination. Data are presented as mean values with error bars indicating SEM. An unpaired two-tailed T-test was used for comparison p = 0.0334.
